# Case Report: Rhabdomyolysis in aneurysmal subarachnoid hemorrhage: A rare case with implications for management

**DOI:** 10.3389/fsurg.2022.1028093

**Published:** 2023-01-06

**Authors:** Gang Liao, Kang Chen, Jiang Xu, Chunliang Wang

**Affiliations:** Department of Neurosurgery, First Affiliated Hospital of Nanchang University, Nanchang City, China

**Keywords:** rhabdomyolysis, cerebral aneurysm, subarachnoid hemorrhage, postoperative, case

## Abstract

**Background:**

In recent years, some cases of rhabdomyolysis after surgery have been reported. In this report, we present an adult patient with rhabdomyolysis after intracranial aneurysm surgery.

**Case Report:**

A 59-year-old male suffered from a coma, fever, and soy sauce urine after intracranial aneurysm clipping. A routine blood examination showed that liver and kidney function were impaired, and creatine phosphokinase(CK) and creatine phosphokinase isoenzyme(CK-MB) levels increased. Therefore, we consider patients with rhabdomyolysis after intracranial aneurysm surgery. A series of treatment schemes, such as intravenous fluid infusion, alkalized urine, and hemodialysis, were adopted immediately, and finally the patient was discharged safely.

**Conclusion:**

For some postoperative patients, once the level of CK/CK-MB increases, acute renal damage occurs, and the urine color turns soy sauce, we should be alert to postoperative rhabdomyolysis.For those patients who have been diagnosed with rhabdomyolysis, we need to take timely treatment measures to avoid an unfortunate occurrence.

## Introduction

Rhabdomyolysis is the breakdown of skeletal muscle integrity with the direct release of intracellular contents including myoglobin, creatine kinase (CK), lactate dehydrogenase, and electrolytes. Common causes include trauma, weariness, strenuous exercise, convulsions, hypothermia, malignant hyperthermia, infections, electrolyte imbalances, medications, toxins, and genetic anomalies (1). The severity of rhabdomyolysis has been upgraded from myoglobin urine, which can lead to acute kidney injury (AKI) to other serious systemic complications. Early recognition and management of this condition are key to reducing permanent morbidity and mortality (2).

Rhabdomyolysis following surgery is a recognized complication that might be brought on by the patient's position, an extended procedure, or certain postoperative risk factors, with numerous reports detailing its impact on the management of the common indications for which patients undergo surgery (3, 4). Aneurysmal subarachnoid haemorrhage(aSAH) is a relative reason for prolonged surgery.

Aneurysmal subarachnoid hemorrhage results in severe disability and mortality in 50% of cases (5). Treatment of the ruptured aneurysm and prevention of delayed rupture of the SAH are important aspects of management to reduce poor outcomes. Endovascular embolization and microsurgical clip occlusion are the main methods to treat ruptured aneurysms. Occasionally, prolonged treatment times lead to patients remaining immobilized on an operating table with the risk of skeletal muscle injury and possibly rhabdomyolysis (6). The treatment of this complex condition can have an impact on SAH complications such as vasospasm, cerebral oedema, seizures, electrolytes, and cardiopulmonary disturbances.

We present a case of rhabdomyolysis following surgically managed aSAH and share lessons from our management of this case.

## Case report

### Patient information

A male patient in his fifties presented with a sudden onset of confusion and syncope. There were no co-morbid medical conditions or family histories of concern.

### Clinical findings

The GCS score was 15, bilateral pupils were equal in size, 2.0 mm in diameter, slow in response to light, neck resistance, and normal muscle strength and muscle tension of the limbs. According to the general assessment, there is no evidence of muscle injury or visceral injury.

### Diagnostic assessment

Head CT showed Fisher II SAH, and cerebral angiography showed that the left middle cerebral artery M1 had a large aneurysm with a neck width of 7.3 mm, a height of 7.9 mm, and a maximum transverse diameter of 8.5 mm; the left middle cerebral artery M2 had a small aneurysm at the beginning of its lower trunk with a neck width of 2.1 mm, a height of 2.2 mm, and a maximum transverse diameter of 1.5 mm; and the left posterior communicating artery had a neck width of 1.3 mm, a height of 3.1 mm, and a maximum transverse diameter of 2.1 mm. The rest of the cerebral vessels and their branches were well developed. Serum potassium was slightly lower (3.31 mmol/L), and other biochemical and hematological indices were normal.

The patient's symptoms are suspected to be connected to a middle cerebral aneurysm hemorrhage. The aneurysm can be treated using one of two methods: Endovascular treatment or craniotomy clipping. Considering the number, location, and operation cost of aneurysms, patients often choose craniotomies to clamp aneurysms.

Digital subtraction angiography (DSA) was performed to better describe the characteristics of the culprit aneurysm and evaluate the remaining vascular tree. Additionally, DSA is not complicated; it was conducted in a dedicated angiography room, and the patient was transported to the surgical operating room one hour later.

After general anesthesia, the patient took the supine position with his head tilted to the right and chose the left pterional approach. After conventional craniotomy, an aneurysm was clipped at the beginning of the posterior communicating artery, and then the neck of the aneurysm was clipped at the M1 bifurcation and M2 inferior branch of the middle cerebral artery, respectively. Intraoperative indocyanine green fluorography confirmed that the clipping effect of three aneurysms was satisfactory. The operation took 7.5 h, and it was complicated by 500 ml of bleeding caused by an aneurysm rupture during the operation. The intraoperative observation was always stable, with no anesthetic reaction, no adverse reaction of blood transfusion, and prophylactic antibiotics (cefotiam) during the operation. Thoroughly stop the bleeding on the wound, place a gelatin sponge near the artery clamp, and give nimodipine to prevent cerebral vasospasm after the operation, Valproate sodium is used for 72-hour antiepileptic prevention. At the same time, in order to reduce brain tissue metabolism and avoid irritability and other emotions, a small dose of sedative and analgesic drugs was given.

On the first postoperative day, the patient was unconscious with a GCS score of 5 T (E1VTM4), which was considered a symptom of cerebral vasospasm. On the third day following the procedure, the patient's peak body temperature was 40.2 °C. There was no sign of an intracranial infection discovered despite lumbar puncture and cerebrospinal fluid analysis. The liver, kidney, and electrolyte functions were monitored while vancomycin (1,000 mg every eight hours) and meropenem (2 g every eight hours) were administered as an anti-infection therapy. On the fifth day after surgery, his body temperature was 39.2°C, and he developed symptoms of impaired liver and kidney function. The level of alanine aminotransferase(ALT) was 169 U/L, aspartate aminotransferase(AST) was 850 u/L, creatinine was 106 U/L, and urea was 10.23 U/L. respectively. To protect liver and kidney function, we will discontinue the use of vancomycin in favor of imipenem to fight infection and magnesium isoglycyrrhizinate to protect the liver. When the patient's urine turned soy-colored on the sixth postoperative day(January 1st), reexamination revealed CK levels of more than 40,000 U/L and CK-MB 1,017.9 U/L, ALT was 354 U/L, AST was 1,180 U/L, creatinine was 131.4 U/L, and urea was 13.03 U/L (Table 1), which are considered diagnostic for rhabdomyolysis syndrome.

**Table 1:** Monitoring of various indices of patients during hospitalization: ALT (9–50 U/L) AST (15–40 U/L) creatinine (57–97 µmol/L) urea (3.1–8 mmol/L) CK (50–310 U/L) CK-MB (2–25 U/L) Potassium (3.5–5.5 mmol/L).

Data	Time	ALT	AST	Creatinine	Urea	CK	CK-MB	Potassium
12.25–12.26	operation operation	17	21	58.1	4	198	17.4	2.38
12.28	(Day 2)	11	18	95.7	6.2	#	#	3.07
12.30	(Day 4)	21	62	135.2	13.47	#	#	2.38
12.31	(Day 5)	169	850	106	3.65	#	#	3.65
1.1	(Day 6)	354	1,180	131.4	10.23	>40,000	1,017.9	5.2
1.2	(Day 7)	572	546	98	10.86	44,569	22,527	4.4
1.3	(Day 8)	540	822	115	10.15	160,142	989	4.5
1.4	Hemofiltration	422	1,246	123.8	14.05	55,920	439.2	3.76
1.5	(Day 10)	299	649	89	6.56	#	#	2.84
1.6	(Day 11)	259	370	83.5	5.01	10,459	75.9	3.22
1.7	(Day 12)	177	184	94	6.78	3,838	30	2.93
1.8	(Day 13)	148	133	103	13.48	3,063	27	3.23
	End *of* hemodialysis							
1.10	(Day 15)	83	64	112.3	16.57	1,310	56.4	3.37
1.12	(Day17)	67	68	96	13.56	#	#	2.79
1.14	(Day 19)	55	50	97	11.39	757	17.1	3.15
1.19	(Day 24)	33	35	68.5	6.01	#	#	3.71
	Implantation of inferior vena cava filter screen							
1.22	(Day 27)	#	#	#	#	516	20.7	4.49
1.28	(Day 33)	50	35	63.2	5.58	161.8	20.4	3.93
2.4	(Day 37)	60	26	60.6	3.62	#	#	3.21
2.5	Lower vena cava mesh removal and Rehabilitate							

On the sixth day after the operation, when the urine of the patient showed soy sauce color, the CK level exceeded 40,000 U/L. On the 33rd day after the operation, the CK level returned to the normal range, and the creatinine level remained within the normal range.

### Therapeutic intervention

The patient was diagnosed with rhabdomyolysis on January 1st. The creatine kinase level of the patient was still high after three days of alkalization urine, active rehydration, and diuresis. Then, from January 4th on, he received hemofiltration treatment once a day. On the 12th day after the operation (January 7th), The consciousness of the patient was better than before, and the GC score was 8 T (E3VTM5), urine color began to fade, creatine kinase level decreased obviously, and successfully disconnected from the ventilator. and symptomatic treatment such as alkalization liquid, infection treatment, renal function protection and nutritional support were continued. On the 13th day after the operation (January 8th), the biochemical values of CK, CK-MB, ALT, AST, creatinine, and urea in the patient's blood were 3,063 U/L, 27 U/L, 148 U/L, 133 U/L, and 13.48 U/L, respectively, so it was decided not to undergo hemodialysis. On the 19th postoperative day (January 14), the patient was conscious with a GCS score of 9 T (E4VTM5), a morning temperature of 38.5 C, CK value of 757 U/L, CK-MB value of 17.1 U/L, ALT of 55 U/L, AST of 50 U/L, creatinine of 97 U/L, and urea of 11.39 U/L, and decided to transfer out of the intensive care unit for follow-up treatment.

### Timeline

You can see the timeline in Table 1.

### Follow-up and outcomes

On January 15th, the patient was transferred to the general ward for further treatment. On January 19th, the patient was conscious with a GCS score of 10 T (E4VTM6) and no fever symptoms. Because of multiple venous thromboses in the right lower limb, the inferior vena cava filter was implanted. The patient can speak after suturing his tracheotomy on January 30th. On February 5th, the patient was conscious (GCS-15 points). After taking out the inferior vena cava filter, he was discharged from the hospital. When the patient was discharged from the hospital, there were no other sequelae, such as headache, nausea, blurred vision, seizures, etc.

## Discussion

This report presented a case of postoperative cerebral aneurysm with cognitive disturbance, rhabdomyolysis, and severe liver and renal failure. Fever and liver and kidney function impairment are the first signs, followed by symptoms of soy sauce-colored urine. Adding adequate liquid, empirical anti-infection treatment, alkalizing urine, preserving liver and renal function, and hemodialysis were all beneficial treatments.

Rhabdomyolysis can be caused by a variety of factors, however postoperative rhabdomyolysis is an uncommon condition whose causes may be connected to obesity, operation duration, and operation posture (7, 8). Patients' poor posture and inadequate cushioning in the operation room might result in skeletal muscle crush damage and hypoxia. Patients with prolonged operation times and obesity are more susceptible to sustaining such injuries, which can lead to rhabdomyolysis following surgery (6). Our patient was not overweight, but the long-term supine position exposes the muscle groups to crush-related injuries.

Hypokalemia is a risk factor for rhabdomyolysis (9). Ischemia might be one explanation for the link between hypokalemia and rhabdomyolysis. It has long been reported that once hypokalemia begins in dogs, muscle blood flow is severely compromised (10). A suitable potassium concentration causes blood vessels in muscles to expand, boosting regional blood flow. Muscles are in relative ischemia when hypokalemia develops, and continuing in this state for an extended period of time can lead to muscular necrosis and rhabdomyolysis. Our patients were found to have hypokalemia during the perioperative period. Although he received potassium ion supplements on time, it is possible that this was a factor in his rhabdomyolysis. Correcting hypokalemia as soon as possible may be an effective way to avoid postoperative rhabdomyolysis.

Infection has been associated with rhabdo in the literature (11–13). The etiology of this association is not well established but is believed to be related to the toxic effect of bacterial metabolites on muscle tissue as well as damage from fever and hypoxia (14). Our patient remained pyrexial for a prolonged period despite antibiotic use.

Prolonged immobilization due to impaired consciousness, cerebral vasospasm, delayed cerebral ischemia, or as part of management may all increase the risk of rhabdomyolysis in patients with aSAH. Good nursing care and vasospasm prophylaxis are also important in avoiding this complication.

A combination of pre-operative hypokalemia, prolonged surgery, persisting post-operative fever, and depressed consciousness were thought to have led to the development of rhabdomyolysis in the present case. During this time, we avoided hypovolaemia to avoid vasospasm and made sure electrolytes were replaced to keep seizures from exacerbating the condition. In managing this patient's fluid resuscitation, urine alkalinization, and hemofiltration as guided by renal function, it was safe.

## Conclusions

Rhabdomyolysis is a serious potential complication of cerebral aneurysm surgery. For patients with hypokalemia before the operation, prolonged operation time, persistent fever after the operation, and cerebral vasospasm leading to decreased consciousness, special attention should be paid to prevent this complication. For patients diagnosed with rhabdomyolysis, we should actively rehydrate intravenously, alkalize urine, protect liver and kidney functions, and conduct hemodialysis when necessary to avoid irreparable consequences for patients.

## Data Availability

The original contributions presented in the study are included in the article/Supplementary Material, further inquiries can be directed to the corresponding author/s.
